# Supplementary material for: Prediction and validation of protein intermediate states from structurally rich ensembles and coarse-grained simulations
Source: Nat Commun. 2016 Aug 31;7:12575. doi: 10.1038/ncomms12575 (PMC5013691; doi:10.1038/ncomms12575)
Supplement: Supplementary Information — Supplementary Figures 1-11, Supplementary Tables 1-8, Supplementary Note 1, Supplementary Methods and Supplementary References. [file ncomms12575-s1.pdf]

SUPPLEMENTARY FIGURES

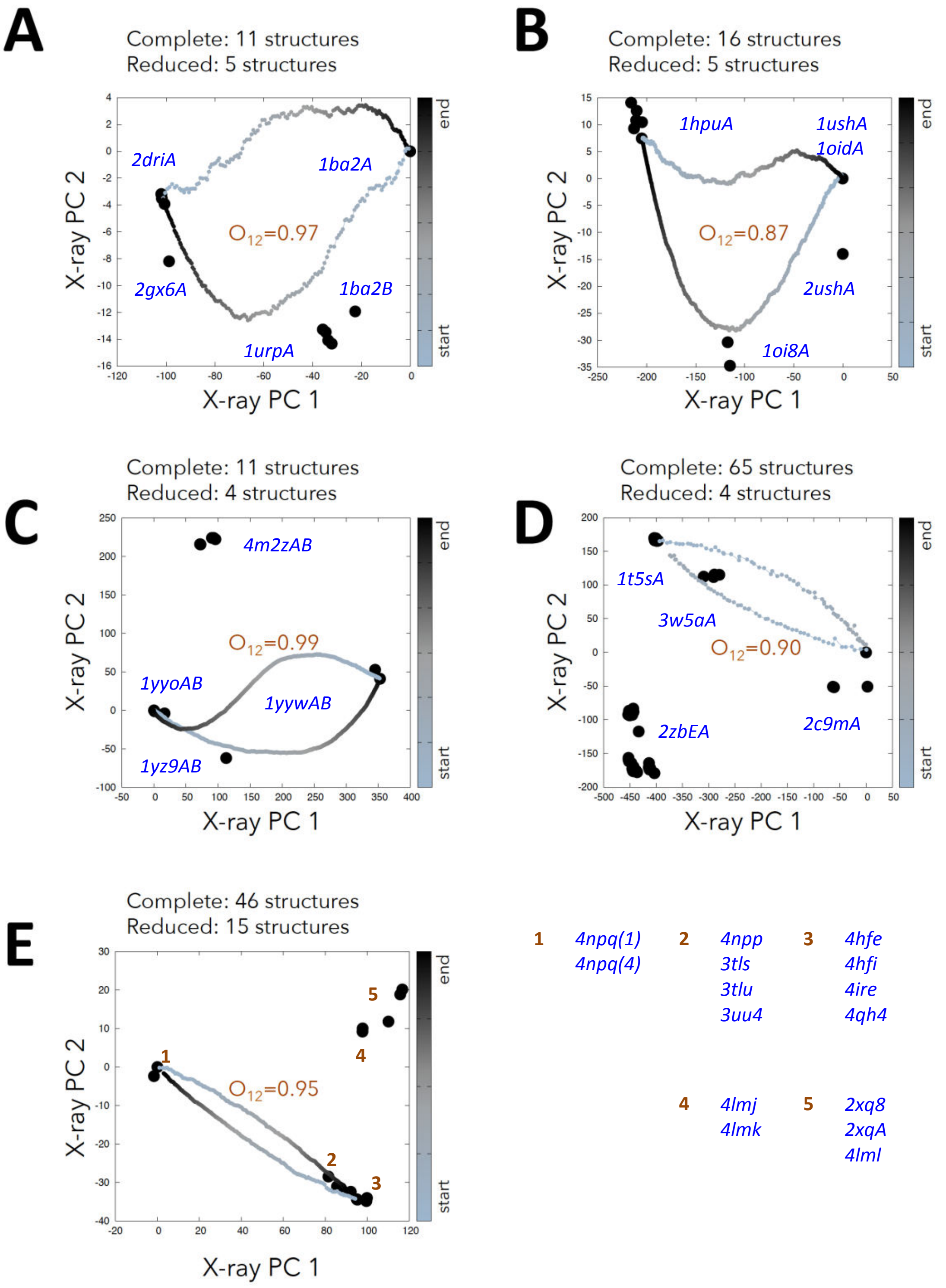

Supplementary Figure 1 | Robustness of the PC1-2 subspace in reduced ensembles. A) RBP; B) 5NTase; C) RNaseIII; D) SERCA; E) GLIC (selected PDBs in blue). The overlap between the first two modes (O_{12}) of the complete versus the reduced ensembles is ≥ 90% with just a few representatives from each cluster, rendering equivalent representations of the conformational space. For SERCA, PCA is reproducible reducing the four E2 sub-clusters to a single one; while GLIC requires a couple of representatives from each cluster to recover both PCs. Ensembles with fewer structures are possible (See overlaps in Supplementary Table 2)

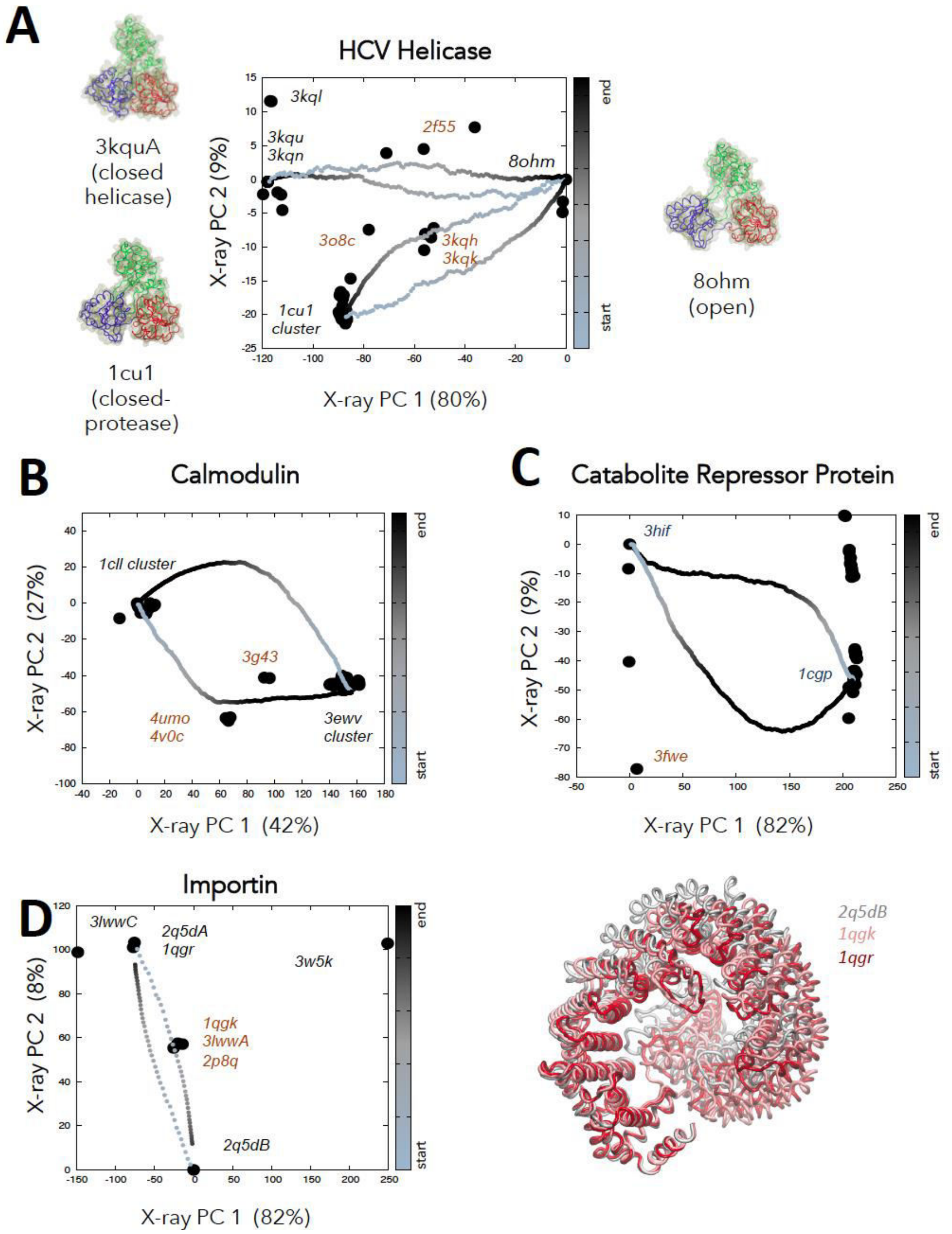

Supplementary Figure 2 | Further examples of multi-state ensembles with possible on-pathway intermediates. For HCV-helicase (A), PC1-2 projections distinguishes the open structures crystallized together with the protease domain (1cu1 and others) from those in which it has been removed (3kqu-like), detecting intermediates already described in the literature (3o8c, 3kqh or 3kqk) and others not characterized as such (mutant 2f55). Similarly happens with Calmodulin, the Catabolite Repressor protein or Importin, with structures that appear as possible transient states between end-conformations that have been stabilized upon binding to different ligands. Further details in Supplementary Table 3.

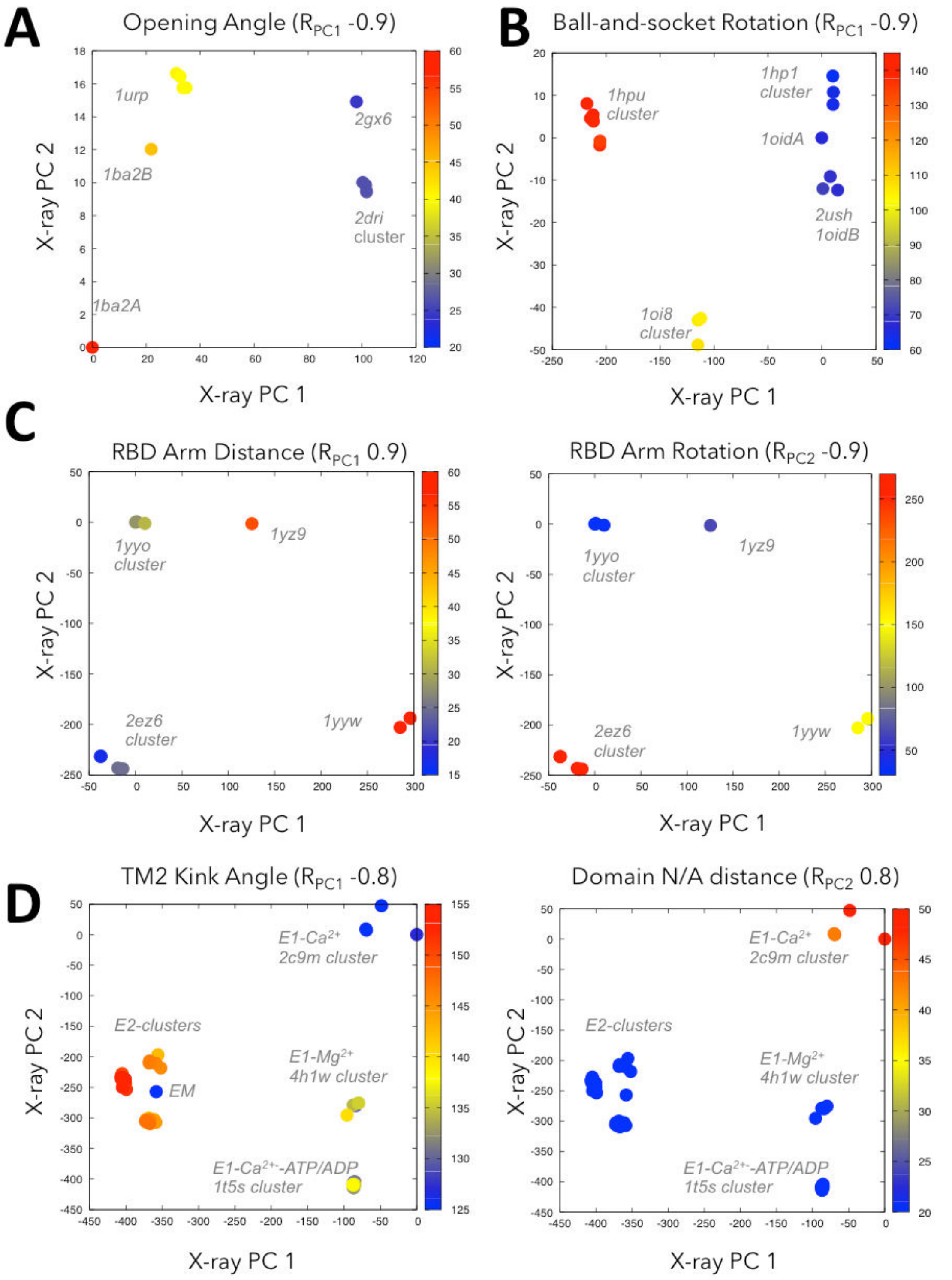

Supplementary Figure 3 | Correlation between major PCs and system-defined heuristic structural variables. A) RBP opening angle; B) 5′-NTase ball-and-socket rotation; C) RNaseIII RBD arm distances (left) and their rotation versus the catalytic cleft (right); D) SERCA TM2 kink (left) and headpiece closure (right); the outlier for TM2 kink is the 1KJU electron-microscopy structure in the E2-clusters (in blue). Angles reported in degrees and distances in Ångstroms. Definitions in Supplementary Methods.

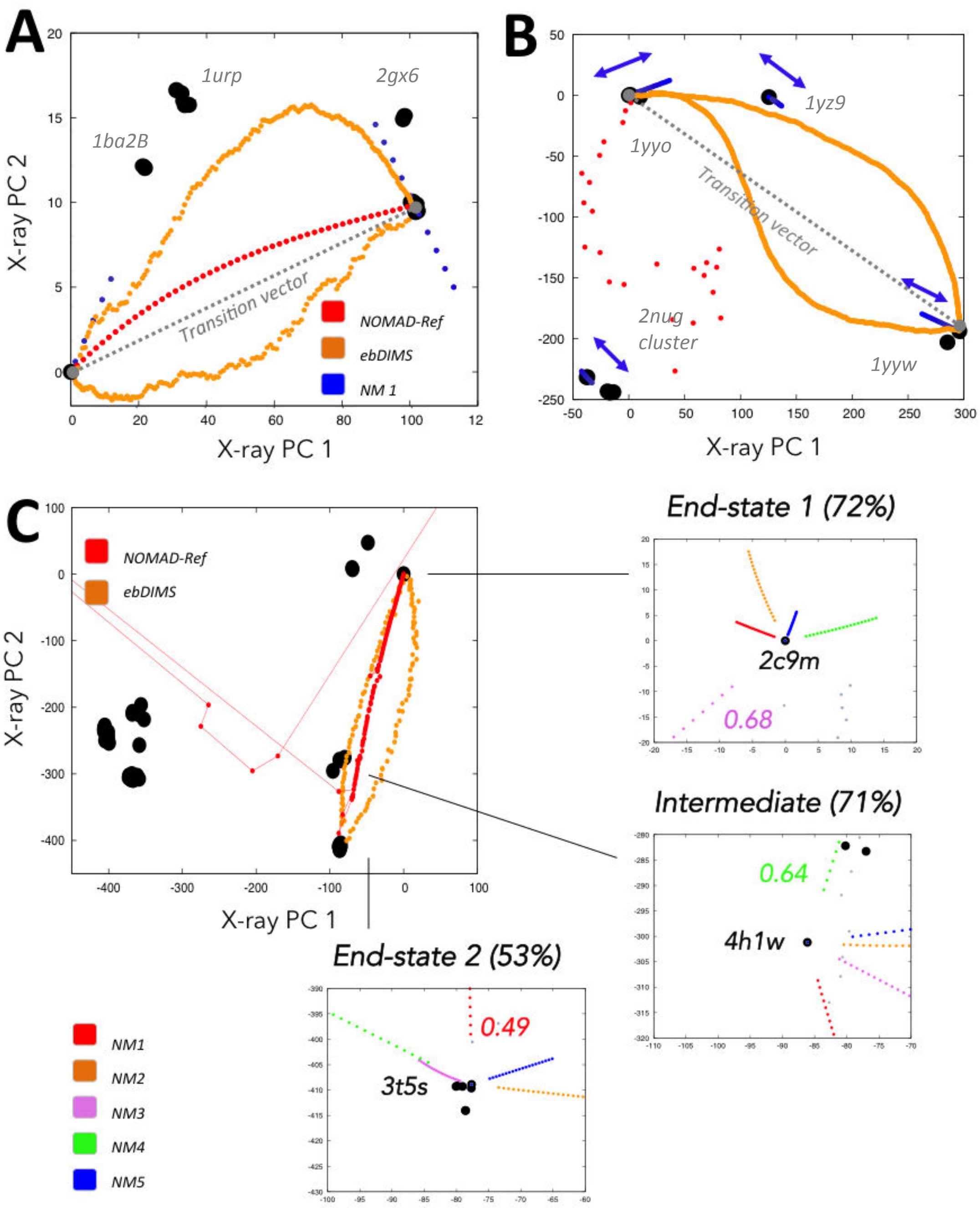

Supplementary Figure 4 | Projections of the first normal mode (NM1) along the pathway for RBP (A) and RNaseIII (B) and SERCA (C) compared with eBDIMS (orange) and NOMAD-Ref (red) pathways. For RBP (left), NM1 aligns perfectly with the distribution of crystal intermediates (1BA2B, 1URP and 2GX6) although it is not the best overlapped with the transition vector, which is followed closely by NOMAD-Ref. A similar situation arises with the large-scale RNaseIII change (right), where normal modes have a low overlap with the transition but nevertheless accurately sample the experimental pathways; in this case, the modes overlapping with the transition lead to an opposite direction from the on-pathway intermediate, 1YZ9, and cannot reach the target 1YYW; for SERCA, at the intermediate state NMs split in the transition directions of the open-close change (aligned with PC2) and the orthogonal pointing to E2 (along PC1) (See overlaps in Supplementary Table 7).

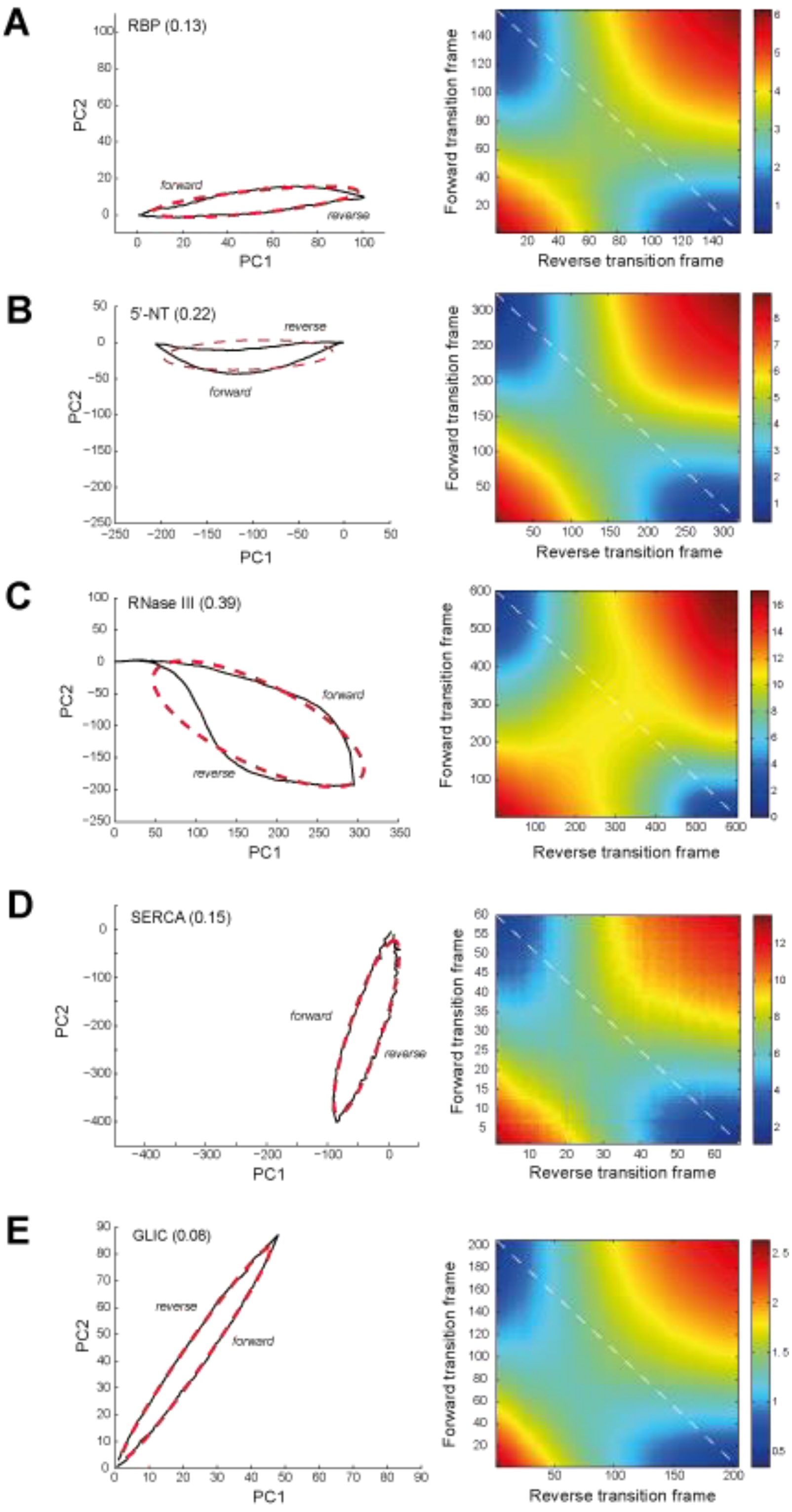

S

Supplementary Figure 5 | Forward and Reverse Pathway asymmetry in the PC1-2 space as measured by ellipsoid eccentricity (left) and contour plot of the rMSD between the trajectories (right). Pathway asymmetry for non-linear methods can be estimated as the eccentricity of an ellipsoid (in red), fitted to the area defined by the forward and reverse paths in the PC1-2 space; values range from 0 (identical pathways) to 1 (totally divergent). Further details in Supplementary Methods.

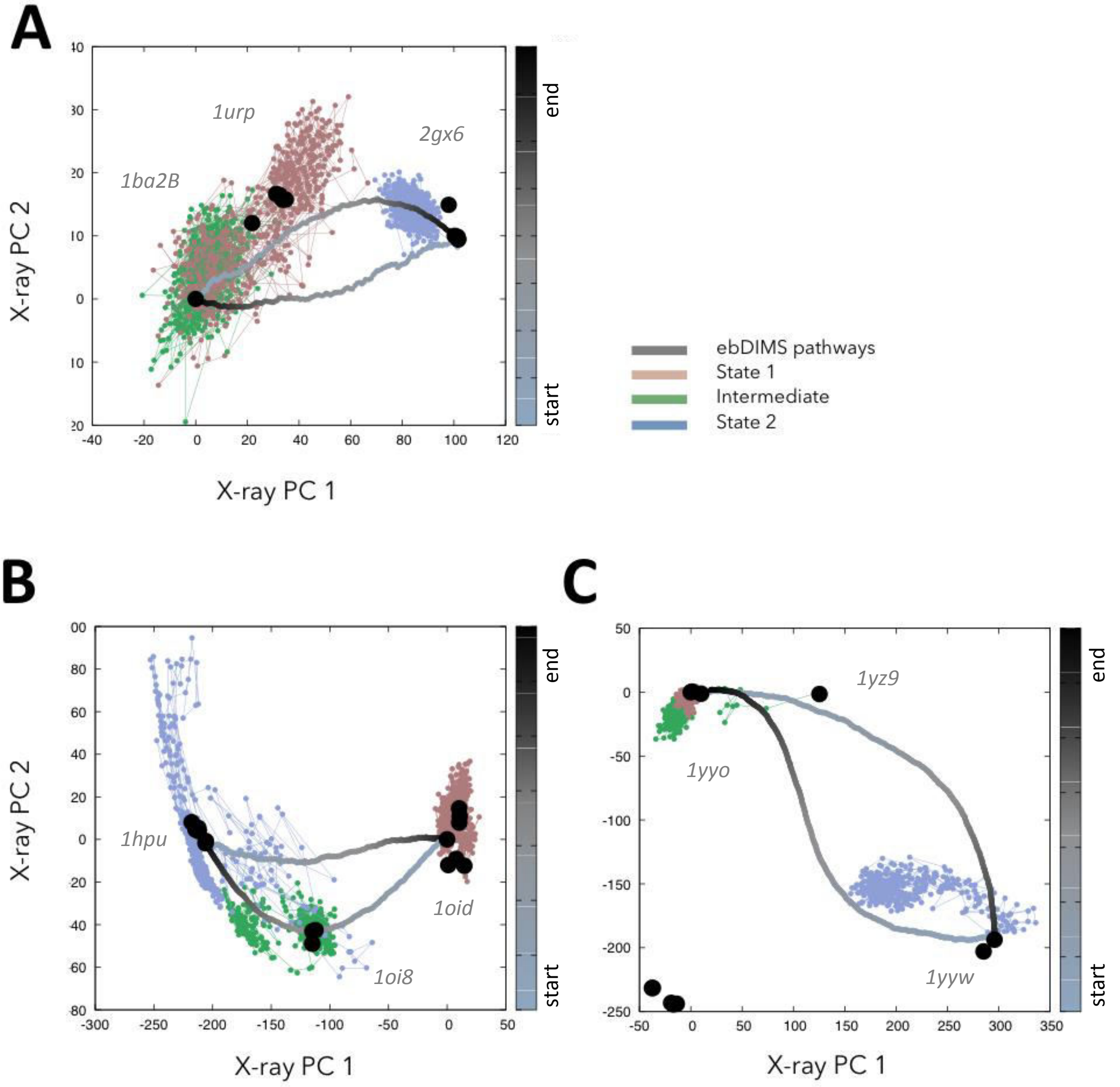

Supplementary Figure 6 | Unbiased MD simulations from the unbound end and intermediate states for A) RBP; B) 5-NTase; C) RNaseIII. For RBP (A), 500ns simulations from the unbound open form (state 1) overlap with eBDIMS forward path sampling spontaneously the partially closed intermediates; the closing trajectory (Fig.9A) follows a similar path sampling a wider area enclosed by the reverse path. Spontaneous sampling for 5-NTase (B) upon removal of the ligand samples half of the eBDIMS area reaching the intermediate 1OI8; for RNaseIII (C), the unbound intermediate 1YZ9 spontaneously transitions to State 1 (1YYO) following the eBDIMS forward path, while unbound 1YYW samples the reverse route. See details of the simulations in Supplementary Table 8 and Supplementary Methods.

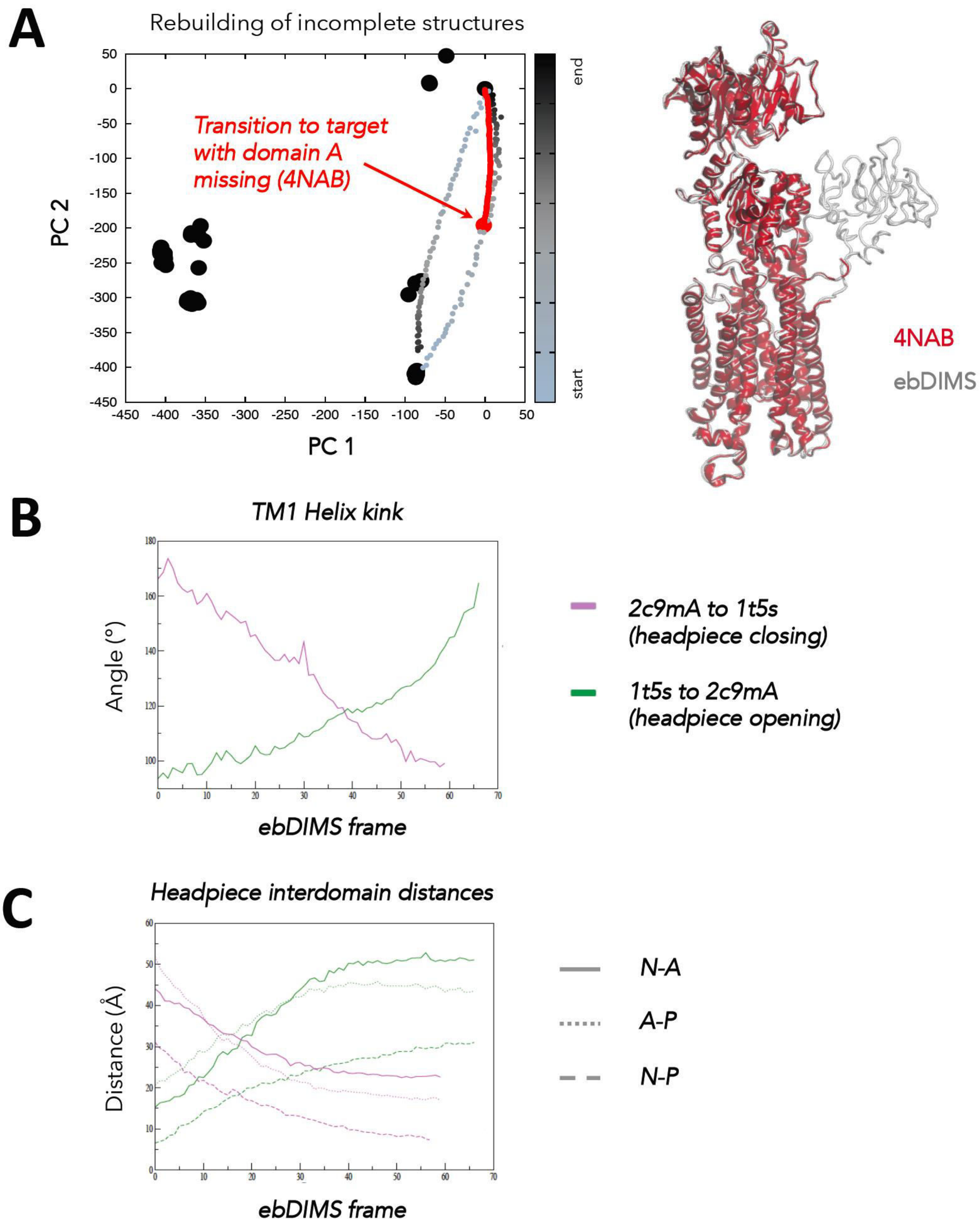

Supplementary Figure 7 | SERCA conformational change. A) Reconstruction of the 4NAB structure starting from 2C9M. eBDIMS can approximate the position of the missing domain-A using as target the incomplete structure 4NAB (right); note that upon reconstruction and projection (left), this structure appears as a possible topological intermediate in the 1T5S-2C9M reverse pathway. B) and C) Evolution of key structural parameters along the open ↔ closed transitions of SERCA: B) Kink Angle for TM1. C) Interdomain distances for the extracellular headpiece. See Supplementary Methods for definitions.

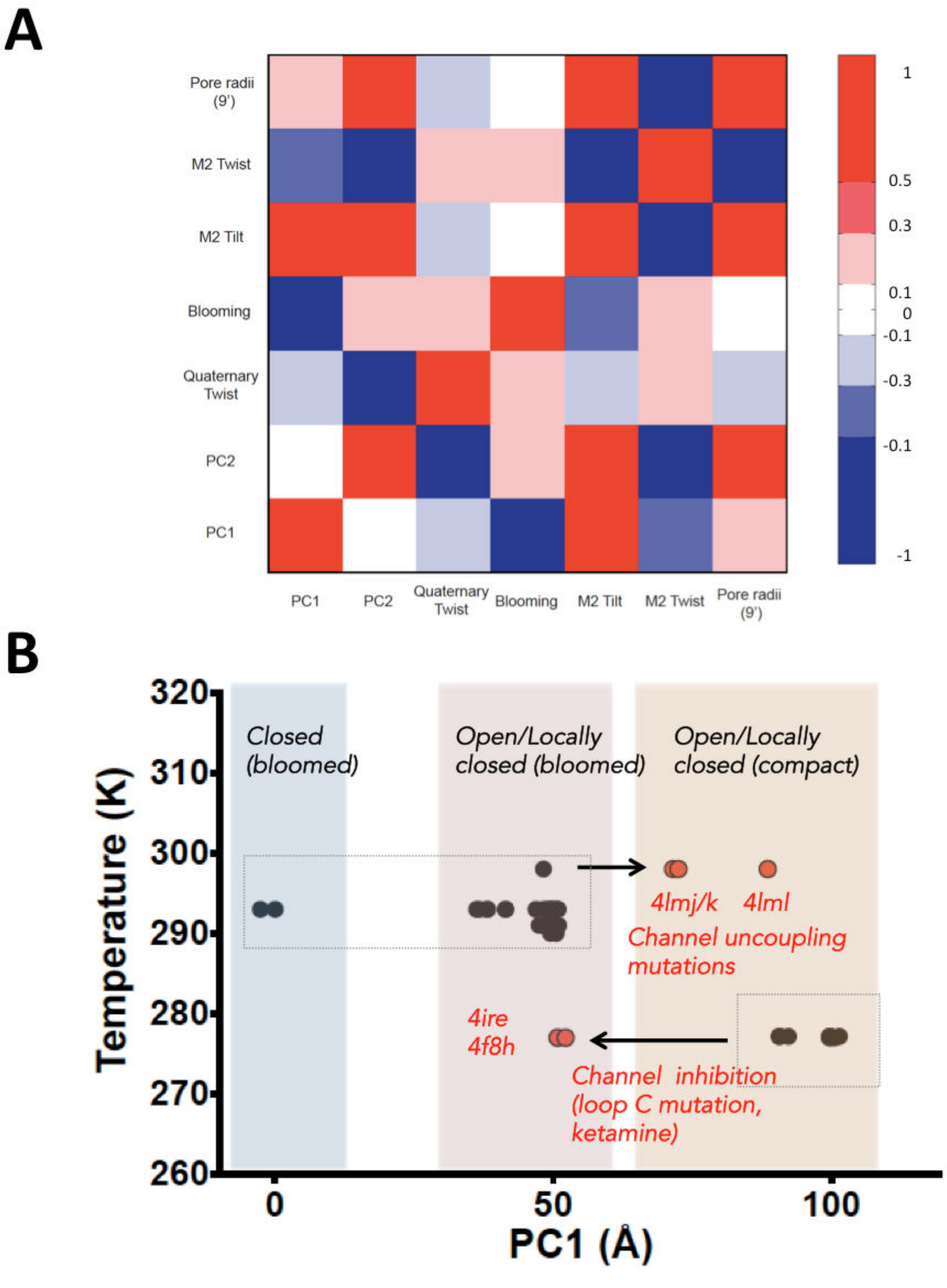

Supplementary Figure 8 | GLIC Principal Components. A) Correlation between GLIC key structural parameters and PC1-2. The analysis of the ensemble also reveals how different functional motions are coupled to each other. B) Correlation between PC1 and crystallization temperature. The only five outliers in the ensemble (46 structures) contain mutations and molecules intended to switch channel activity. See Supplementary Methods for definitions of GLIC heuristic variables.

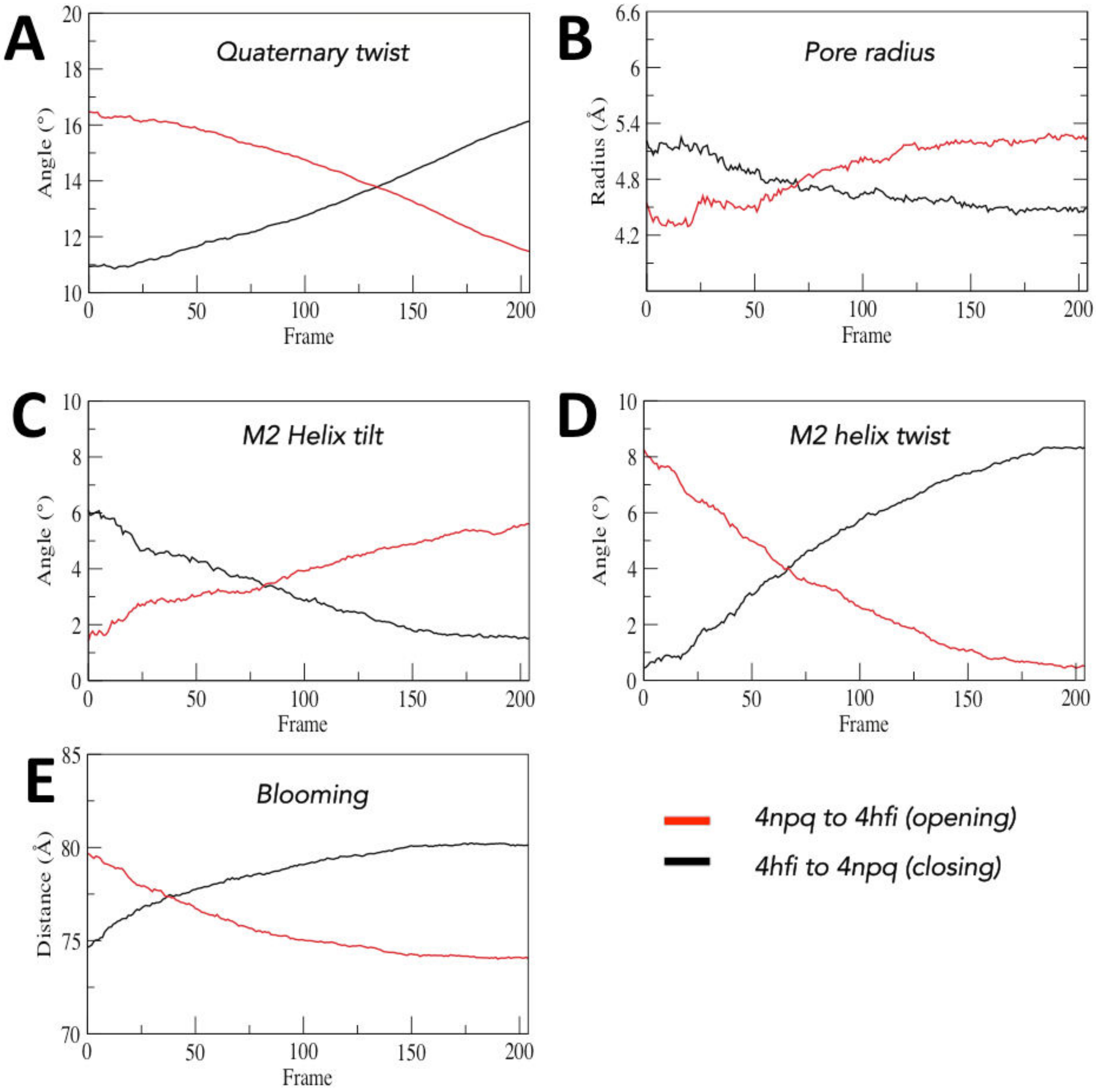

Supplementary Figure 9 | Evolution of key structural parameters for GLIC gating transitions. A) Quaternary twist, B) Pore radius, C) M2 helix tilt, D) M2 helix twist, and E) Blooming along the opening (red) / closing (black) GLIC eBDIMS trajectories. Note that, in the opening transition, un-blooming (E) precedes the change in quaternary twist (A) as suggested in the literature, and the opposite happens for the closing one. See Supplementary Methods for definitions.

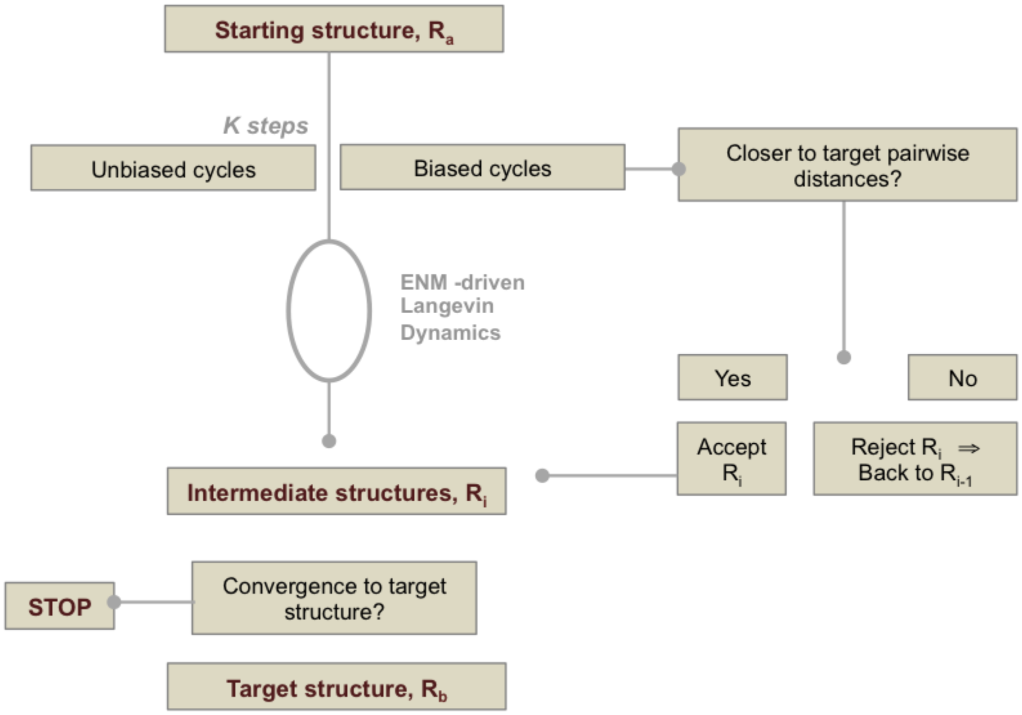

Supplementary Figure 10 | Flowchart of the basic eBDIMS algorithm. Starting structure moves in an Overdamped Langevin simulation and every k steps pairwise distances are checked; if the current intermediate conformer, R_i, is approaching the known pairwise distances of the target is accepted or otherwise rejected. Iteration proceeds until convergence with the target basin is achieved. See Methods for further details.

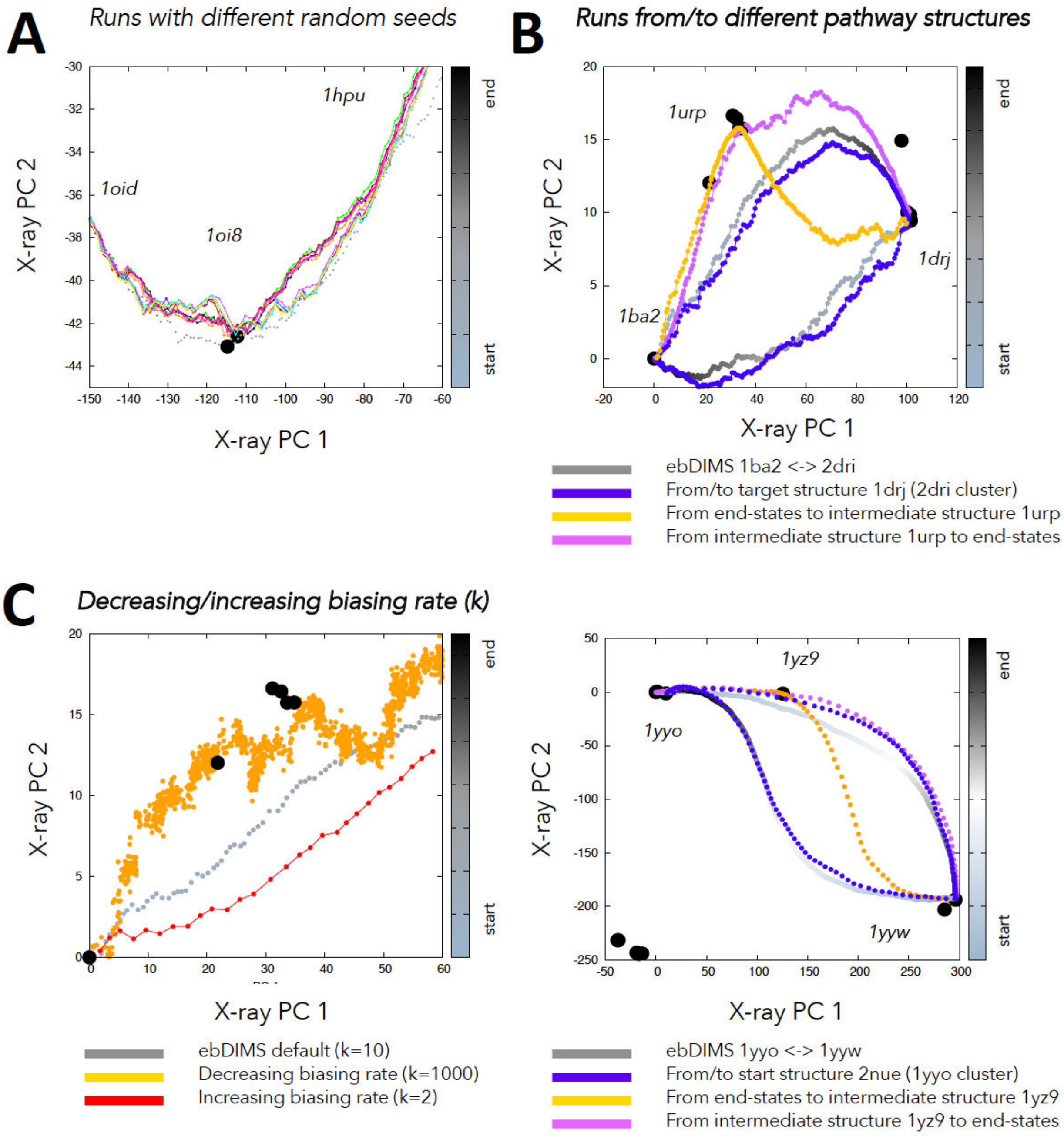

Supplementary Figure 11 | Convergence of eBDIMS trajectories with default parameters and effect of the biasing strength on the sampling-width. A) Trajectories run with different random seeds; B) Trajectories from/to different structures along the pathway for RBP (above) and RNaseIII (below); note that although the trajectories are slightly different each time, they tend to converge sampling the same region. C) Trajectories run increasing or decreasing biasing frequency (k); decreasing the bias to better sample intermediate states can enhance the sampling width, while increasing it speeds up calculations but tends to render trajectories closer to Cartesian-like straight interpolations.

SUPPLEMENTARY TABLES

Supplementary Table 1 | Model proteins and end-/intermediate- states description

Name	State1	State2	Motion	$RMSD_t$	N-mer	Heuristic Intermediates	Topological Intermediates
**RBP**	1BA2(A) (21.4)<br>Open<br>Unbound	2DRI (19.8)<br>Closed<br>Ribose-bound	*Hinge*	6.2	1-mer	1BA2(B) and 1URP[1]	2GX6
**5'-NTase**	1OID (21.53)<br>-<br>Unbound	1HPU(A) (21.59)<br>-<br>Nucleotide-bound	*Rotation*	9.3	1-mer	1OI8[2]	4WWL
**RNaseIII**	1YYO (24.5)<br>Closed<br>dsRNA-bound	1YYW (26.6)<br>Open<br>dsRNA-bound	*Complex*	17.8	2-mer	1YZ9[3]	2EZ6,<br>2NUF, 2NUG<br>4M2Z, 4M30
**SERCA**	2C9M (38.6)<br>Open headpiece<br>Ca^{2+}-bound	1T5S (37.7)<br>Closed headpiece<br>Ca^{2+}/Nucleotide-bound	*Complex*	14.16	1-mer	Not described	4H1W,<br>3W5A, 3W5B[4]<br>4NAB
**GLIC**	4NPQ (37.6)<br>Open ECD<br>Closed pore<br>Resting (pH=7.5)	4HIF (37.2)<br>Closed ECD<br>Open pore<br>Conducting (pH=4)	*Complex*	2.6	5-mer	3TL* and 3UU*[5]<br>4NPP[6]<br>*"Bloomed"*	4LMJ and 4LMK<br><br>*"Compact"*

State 1 = Starting state (Reference); Radius of gyration in parenthesis
State 2 = Target state; R_g in parenthesis
$RMSD_t$ = RMSD between end-states
N-mer = Number of Chains

1. Björkman, J. & Mowbray, S. L. Multiple open forms of ribose-binding protein trace the path of its conformational change. *J. Mol. Biol.* **279,** 651–664 (1998).
2. Schultz-Heienbrok, R., Maier, T. & Sträter, N. Trapping a 96 degrees domain rotation in two distinct conformations by engineered disulfide bridges. *Protein Sci.* **13,** 1811–1822 (2004).
3. Gan, J. *et al.* Intermediate states of ribonuclease III in complex with double-stranded RNA. *Structure* **13,** 1435–1442 (2005).
4. Smolin, N. & Robia, S. L. A structural mechanism for calcium transporter headpiece closure. *J. Phys. Chem. B* **119,** 1407–1415 (2015).
5. Prevost, M. S. *et al.* A locally closed conformation of a bacterial pentameric proton-gated ion channel. *Nature Structural & Molecular Biology* **19,** 642–649 (2012).
6. Sauguet, L. *et al.* Crystal structures of a pentameric ligand-gated ion channel provide a mechanism for activation. *Proc. Natl. Acad. Sci. U. S. A.* **111,** 966–71 (2014).

Supplementary Table 2 | Structural ensembles features and PC12-space robustness

Name	N_{res}	N_e	$RMSD_e$	Var	V_{PC1}	V_{PC2}	V_{PC1-2}	N_{clust}	N_{red}	O_2	O_{11}-O_{22}
**RBP**	271 (1-271)	11	3.7±2.4	1505	97%	2%	99%	5	2	0.50	0.99-0.00
			(6.2)						3	0.99	0.99-0.97
									5	0.97	0.99-0.94
**5'-NTase**	523(26-548)	16	5.0±3.8	7521	95%	4%	99%	4	2	0.50	0.99-0.00
			(9.6)						3	0.98	0.98-0.94
									5	0.87	0.99-0.75
**RNaseIII**	432(3-218)	11	9.6 ±5.8	27867	51%	43%	97%	4	2	0.50	0.69-0.00
			(17.1)						3	0.71	0.64-0.28
									4	0.99	0.80-0.80
**SERCA**	994(1-994)	65	14.1 ± 3.1	30474	58%	26%	84%	7	2	0.46	O_{12}=0.88
			(15.7)						2	0.43	0.62-0.00
									3	0.48	O_{12}=0.87
									3	0.92	$O_{12/21}$=0.60/0.57
									4	0.90	0.60-0.60
**GLIC**	1540(8-315)	46	2.7 ± 0.5	1461	42%	29%	71%	5	2	0.46	O_{21}=0.71
			(3.1)						3	0.83	0.45-0.44
									4	0.72	0.49-0.51
									5	0.81	0.44-0.51
									6	0.86	0.60-0.47
									10	0.74	0.80-0.40
									15	0.95	0.54-0.54

N_{res} = Number of residues for the consensus full-length alignment (in brackets, residue indexes of the overlapped regions)
N_e = Number of structures in the ensemble
$RMSD_e$ = RMSD of the ensemble versus reference (reported as AVG + SD; in brackets, maximal rMSD between structures)
Var = Structural variance sampled by the ensemble in Angstroms
N_{clust} = Number of functional clusters in the PC1-2 subspace
N_{red} = Number of structures in the reduced ensemble
O_2 = Cumulative overlap between first two eigenvectors of the full ensemble and the reduced ensemble
O_{11} = Overlap between the first eigenvectors of the full ensemble and the reduced ensemble
O_{22} = Overlap between the second eigenvectors of the full ensemble and the reduced ensemble
See Supplementary Methods for overlap definitions.

Supplementary Table 3 | Further multi-state ensembles with potential intermediates

Name	State1	State2	Motion	$RMSD_t$	N-mer	Nres	Nens	V_{PC1}-V_{PC2}	eBDIMS-PCA intermediates
**HCV helicase**	8OHM(A)	3KQU	*Rotation*	5.6	1-mer	435	45	80%-9%	2F55
		1CU1		4.4					3KQH and 3KQK*[7] 3O8C*[8]
**Calmodulin**	1CLL	3EWV	*Hinge*	14.8	1-mer	134	44	42%-27%	3G43, 4UMO, 4V0C
**Catabolite Repressor Protein**	3HIF	1CGP	*Complex*	11	2-mer	380	34	82%-9%	3FWE
**Importin**	2Q5D(B)	2Q5D(A)	*Wrapping*	4.7	1-mer	859	8	82%-8%	1QGK, 3LWW(A), 2P8Q

** Heuristic or literature-known intermediates with references:*

7. Gu, M. & Rice, C. M. Three conformational snapshots of the hepatitis C virus NS3 helicase reveal a ratchet translocation mechanism. *Proc. Natl. Acad. Sci. U. S. A.* **107,** 521–528 (2010).
8. Appleby, T. C. *et al.* Visualizing ATP-dependent RNA translocation by the NS3 helicase from HCV. *J. Mol. Biol.* **405,** 1139–1153 (2011).

Supplementary Table 4 | Summary of path-sampling methods tested.

Level	Name*	Potential/ Force-field	Solvent	Linear	Function/Biasing$	Details
**All-atom**	**Morph**	CHARMM/ XPLOR		Yes	Cartesian interpolation + Energy minimization	http://www2.molmovdb.org/wiki/info/index.php/Morph_Server
	**NMSim**	FIRST decomposition + RCNMA + Structural refinement		No	NM biasing + stereochemical constraints	http://cpclab.uni-duesseldorf.de/nmsim/
	**Climber**	ENCAD force-field	-	No	Elastic restraint + Pulling of interresidue distances	https://simtk.org/projects/climber
**C-alpha**	**NOMAD-Ref**	Tirion's ENM		Yes	NM interpolation (Kim's algorithm)	http://lorentz.dynstr.pasteur.fr/trajectories/trans_submission.php
	**MinActionPath**	Two-ENMs	Overdamped Langevin	Yes	Minimum OM action (2ODEs + BCs)	http://lorentz.dynstr.pasteur.fr/joel/traj_newpath_submission.php •
	**iENM**	Double-well ENM		Yes	NM interpolation + Collision Penalty	http://enm.lobos.nih.gov/start_ienm.html
	**iMODS**	Internal coordinates ENM		No	NM interpolation in dihedral angle space	http://imods.chaconlab.org
	**eBDIMS**	ED-ENM	Overdamped Langevin	No	Brownian Dynamics + DIMS (interresidue distances)	

** See References in Methods section*

$ Abbreviations: OM = Onsager-Machlup minimal action; ODEs = Ordinary Differential Equations; BCs= Boundary Conditions

Supplementary Table 5 | Minimal C-alpha rMSD versus PC1-2 distances to representative intermediates and target structures. For each cell, rMSD in 1st line and PC distance in 2nd. Target structures in forward and reverse paths defined in Supplementary Table 1. Definition of PC distance in Methods.

Method	Trajectory Direction	RBP 1urp/target	5'-NTase 1oi8/target	RNaseIII 1yz9/target	SERCA 3w5a/target	GLIC 3tls/target
**NMSim**	forward	0.91/0.57	2.24/3.11	5.03/0.99	4.61/3.61	1.97/2.11
		6.82/0.12	10.10/30.08	52.34/0.09	40.00/3.99	18.25/35.48
	reverse	1.19/1.61	1.96/2.02	7.52/2.74	4.53/5.83	1.24/2.01
		4.83/7.08	11.39/5.94	61.25/5.21	9.51/60.13	1.40/26.57
**Climber**	forward	1.23/0.63	1.62/0.60	4.49/0.50	5.40/0.44	1.10/0.37
		6.72/4.12	4.21/9.43	13.96/1.81	1.37/7.42	0.07/7.55
	reverse	1.88/0.62	2.53/0.66	5.77/0.58	10.30/0.47	2.59/0.49
		13.16/3.35	27.95/2.05	51.64/0.83	38.66/1.75	1.08/14.62
**NOMAD-Ref**	forward	0.88/0.00	1.54/0.00	7.11/0.00	3.33/0.00	1.02/0.00
		10.53/0.00	6.62/0.00	123.68/0.00	20.11/0.00	2.25/0.00
**MinActionPath**	forward	0.77/0.00	1.59/0.00	4.40/0.00	3.65/0.00	1.02/0.00
		9.19/0.00	8.85/0.00	12.72/0.00	21.90/0.00	1.23/0.00
**iENM**	forward	0.55/0.04	3.03/0.40	7.17/16.13	4.88/0.57	1.02/0.00
		2.32/0.03	11.55/1.92	125.43/242.22	9.94/6.94	0.91/0.62
**iMODS**	forward	0.60/0.44	3.30/1.21	5.12/1.03	4.50/1.78	1.19/1.03
		6.64/0.88	9.97/5.49	53.67/0.96	33.63/9.28	2.59/19.85
	reverse	1.27/0.00	2.96/1.58	7.66/2.73	4.70/2.05	1.05/1.18
		6.19/0.05	19.70/4.95	105.51/9.43	5.49/7.84	7.90/25.61
**eBDIMS**	forward	1.47/0.09	1.84/0.16	4.11/0.46	4.58/1.89	1.05/0.28
		5.02/0.61	0.55/1.61	10.70/0.51	2.89/33.83	0.63/4.87
	reverse	1.70/0.08	2.71/0.14	5.78/0.18	3.74/1.07	1.07/0.24
		15.63/0.48	32.16/0.64	50.92/0.30	48.77/13.25	3.07/3.25

Supplementary Table 6 | Overlaps between collective PCs and the vector of the transitions

Name	State 1	State 2	O_{PC1}	O_{PC2}	O_{PC3}	O_{PC4}	O_{PC5}	O_{PC1-2}
**RBP**	1BA2	2DRI	0.994	0.096	0.029	0.007	0.001	0.999
**5'-NTase**	1OID	1HPU	0.994	0.094	0.026	0.035	0.023	0.998
**RNaseIII**	1YYO	1YYW	0.831	0.544	0.089	0.025	0.015	0.994
**SERCA**	2C9M	1T5S	0.131	0.950	0.223	0.055	0.012	0.959
**GLIC**	4NPQ	4HIF	0.464	0.840	0.183	0.081	0.041	0.959

$O_{PC\,N}$ = Overlap of the n-th PC with the transition vector
$O_{PC\,1\text{-}2}$ = Cumulative overlap of the PC1-2 subspace with the transition vector
See Supplementary Methods for overlap definitions.

Supplementary Table 7 | Overlaps of NMs from state 1, intermediate and state 2 forms with the transitions

Name	State 1	O_5	O_{max}	Intermediate	O_5	O_{max}	State 2	O_5	O_{max}
**RBP**	1BA2	0.95	0.50(1)<br>0.77(2)	1URP[1]	0.79	0.75(2)	2DRI	0.92	0.82(1)<br>0.42(3)
**5'-NTase**	1OID	0.69	0.27(1)<br>0.51(2)<br>0.29(4)	1OI8[1]	0.70	0.63(1)<br>0.28(2)	1HPU	0.62	0.55(2)
**RNaseIII**	1YYO	0.58	0.34(1)<br>0.29(2)<br>0.22(3)<br>0.30(4)	1YZ9[1]	0.81	0.66(3)<br>0.40(2)	1YYW	0.64	0.44(1)<br>0.40(2)
**SERCA**	2C9M	0.72	0.68(3)	4H1W[2]	0.71	0.64(4)<br>0.27(1)	1T5S	0.53	0.49(1)
**GLIC**	4NPQ	0.75	0.54(3)<br>0.43(5)<br>0.30(2)	3TLU[1]	0.75	0.55(5)<br>0.45(4)	4HIF	0.77	0.65(5)<br>0.38(4)

O_5 = Cumulative overlap of the first five modes (see definition in Supplementary Methods)
O_{max} = Best overlapped mode
[1] Literature-defined intermediate (see definition in Methods)
[2] Topological intermediate (see definition in Methods)

Supplementary Table 8 | Summary of Molecular Dynamics data

Name	MD scheme	Biasing coordinate	Starting structure	Time	Observed transition	Overlap with eBDIMS	Analyzed trajectories (References)
**RBP**	**Unbiased**	-	1BA2	500ns	Partial closing to 1URP	Partially samples eBDIMS area	-
			1URP	500ns	Partial opening to 1BA2	Partially samples eBDIMS area	
			2DRI	500ns	-	Stays in 2DRI cluster minima	
	**Steered**	Interdomain COM distance	1BA2	5ns	Partial closing to 1URP	Samples eBDIMS path	-
			2DRI	5ns	Full opening to 1BA2	Fully samples eBDIMS area	
	**AWH**	Interdomain COM distance	1BA2	500ns	Not converges	-	
			2DRI	500ns	Full opening to 1BA2	Partially samples eBDIMS area	
**5-NTase**	**Unbiased**	-	1OID	500ns	Partial transition to 1OI8	Partially samples eBDIMS area	-
			1OI8	500ns	Partial transition to 1OID	Partially samples eBDIMS area	
			1HPU	500ns	-	Stays in 1HPU cluster minima	
**RNAseIII**	**Unbiased**	-	1YYO	400ns	-	Stays in 1YYO cluster	-
			1YZ9	400ns	Partial closing to 1YYO cluster	Partially samples ebDIMS forward path	
			1YYW	400ns	Partial closing	Partially samples eBDIMS reverse path	
**SERCA**	**Multi-run**	-	1SU4	400ns (4x)	Partial closing to 4H1W cluster	Partially samples eBDIMS area	Smolin and Robia (2015)[4]
			Intermediates	40ns (6x)	-	Partially samples eBDIMS area	
			2ZBD	400ns (3x)	-	Stays in 1T5S cluster minima	
**GLIC**	**Single long**	-	3EAM	1µs	Full closing to 4NPQ cluster<br>Partial opening to 4HFI	Fully samples eBDIMS area	Bocquet et al (2009)[9]<br>Nury et al (2010)[10]
			4NPQ	500ns		Partially samples eBDIMS area	

4. Smolin, N. & Robia, S. L. A structural mechanism for calcium transporter headpiece closure. *J. Phys. Chem. B* **119,** 1407–1415 (2015).
9. Bocquet, N. *et al.* X-ray structure of a pentameric ligand-gated ion channel in an apparently open conformation. *Nature* **457,** 111–114 (2009).
10. Nury, H. *et al.* One-microsecond molecular dynamics simulation of channel gating in a nicotinic receptor homologue. *Proc. Natl. Acad. Sci. U. S. A.* **107,** 6275–80 (2010).

Supplementary note 1:

SUPPLEMENTARY DISCUSSION

In general, NMA-based methods can approximate efficiently simple non-asymmetric transitions (RBP, 5'NTase, GLIC or SERCA), both in the PC1-2 space and in terms of rMSD (Supplementary Table 5) with little differences among them other than computation speed or stability of the algorithm as seen from projections onto the PC1-2 subspace. Regarding stability in the PC1-2 space, some of the methods (iENM and NOMAD-Ref) displayed jagged trajectories with sudden changes in direction for the most challenging examples (5'NTase, RNaseIII or SERCA), which can be related to distortions along the sampled NMs when approaching bifurcation points (see Supplementary Figure 4 and discussion above). However, a key difference among the tested methods is their ability to provide non-linear paths, covering a wider conformational space by approaching the boundaries of the low-energy troughs sampled by MD simulations. Among all NMA-based methods, MinActionPath (which also uses Langevin Dynamics) yielded the results closest to eBDIMS and Climber in one of the two possible alternative routes, but only iMODS was capable of sampling significantly divergent forward/reverse paths. Notably, iMODS was as well as the fastest of the algorithms tested, providing results in just a few minutes. The notable agreement between the paths explored by NMA methods and the experimentally tracked pathways demonstrates that functional motions are encoded in protein shape, which can be effectively described by elastic network potentials.

SUPPLEMENTARY METHODS

Comparison with path-sampling algorithms. All the methods were run with their default parameters described in the original papers (See *Methods* for references) and webservers/executables (See Supplementary Table 4). The most relevant are reported below:

- ***NMSIM:*** Targeted simulation with RCNMA mode was used, the default cutoff was set to 1Å and the normal mode range was 5. Mutation containing structures were restored to wild type using MODELLER as NMSIM requires matching end states. Due to limitations of the webserver GLIC was run using only the backbone atoms.
- ***Climber:*** transitions were run using a default number of steps of 50.
- ***iMODS*****:** was run using global superimposition, the number of modes chosen was 0.1 (10%), dihedral ls were not frozen and C-alpha RMSD separation between frames was set to 1Å.
- ***NOMAD-Ref:*** the default cutoff value is set to 10Å and the number of steps is 49; for large transitions (SERCA) and poorly linked domains (RNAseIII) values were increased as recommended (100 steps and 15Å)
- ***iENM:*** the default cutoff for the ENM is set to 10Å, and the force constants 10kcal/mol.$Å^2$, with a collision radius of 4Å
- ***MinActionPath:*** the default cutoff value is set to 10Å.

Overlap of subspace motions with the observed X-ray conformational change. The similarity between the motion subspace of the protein (ensemble-defined or computed from ED-ENM (see *Methods*) and the experimental transition is measured as:

$$\alpha_k = \frac{\Delta r \cdot \nu_k}{\|\Delta r\| \|\nu_k\|}$$

Where $\Delta r = (R_2 - R_1) / \left\| R_2 - R_1 \right\|$ is the unitary transition vector between the two sets of coordinates, R_1 and R_2, describing the end-states, and ν_k is the *k-th* NM or X-ray PC. Generalization of above equation for the m-important deformation modes (the minimum set explaining a given threshold of protein motions set i.e. by the variance) yields to a similarity index ranging from 0 (no similarity in the directions of motion) to 1 (perfect similarity):

$$\delta_k = \frac{1}{m} \left(\sum_{k=1}^{m} \alpha_k^2 \right)^{1/2}$$

Calculation of heuristic system-defined structural variables. The heuristic variables were selected taking into account the literature for each system and computed as follows:

- ***RBP:*** the *opening angle* was defined as that between the vectors from the center-of-mass (CM) of both ligand-binding domains to their interface.
- ***5'-NTase:*** the *ball-and-socket rotation* was evaluated aligning the N-terminal domain to the z-axis and computing its angle versus the major axis of the minor domain.
- ***RNaseIII:*** *RBD arm separation* was computed as the distance between their CMs; *RBD arm rotation* was defined as the angle between their major axis and the axis of symmetry of the dimer through the catalytic cleft.
- **SERCA:** The distance between headpiece N, A and P domains were defined as the distance between Ca atoms of residues 171, 489, and 690 respectively. The angle of the kink regions of TM1 and TM2 helices was defined between C-alpha atoms of residues 51, 61, and 75, and 233, 247 and 273, respectively.

- ***GLIC:** Blooming* was evaluated as the maximal radius of the extracellular domain defined by maximally separated residues at the tip of the five subunits. *Quaternary twist* was calculated as the average rotation angle of each subunit with the vector from extracellular domain and transmembrane domain CM to overall CM on the XY plane. The *tilt and the twist angle of M2 helices* were calculated as an average over five subunits. In order to get comparable angles between subunits a reference structure (4NPQ) was aligned to the center of mass of each M2 helix. X-axis was characterized between two centers of mass; M2 helix and the protein center of mass. Z-axis was chosen as the principal of inertia parallel to the symmetry axis. Finally Y-axis was defined as vector normal of XZ plane. With these axes, tilt angle is calculated between the projected helical axis onto XZ plane and Z-axis and twist angle between the projected helical axis onto YZ plane and Z-axis. Pore calculations were performed with HOLE using 0.5Å step from C-alpha atoms only. Pore radius at 9' was averaged over a 2.5Å window in both directions from the center of mass of 9' residues.

Forward and reverse pathways asymmetry. To estimate the divergence between non-linear forward/reverse pathways upon projection in the PC1-2 subspace, the 2D-area delimited by the two paths was fitted by least-squares to an ellipsoid as described in[11] and its eccentricity measured upon implementation in a MATLAB routine. The contour plot of the rMSD between the frames of both forward and reverse trajectories was computed with in-house tools and plotted with MATLAB.

Molecular Dynamics Protocols for RBP, RNAseIII, 5-NTase and GLIC. Crystal structures of RBP (1BA2, 2DRI, 1URP), RNAseIII (1YYW, 1YYO, 1YZ9) and 5-NTase (1OID, 1OI8, 1HPU) were used for molecular dynamics simulations after removing the present ligands. Each structure was prepared individually using Amber99SB-ILDN[12] force field with virtual sites in GROMACS 5.1[13]. RBP and 5-NTase structures were placed in a dodecahedron box while RNAseIII structures were placed in a cubic box. Each system was solvated with TIP3P waters and Na^+ and Cl^- ions were added to neutralize it. Energy minimization was carried out using steepest descent algorithm to a tolerance of 1000 kJ/mol/nm with 1fs timestep. Equilibration was carried out in two phases. First, a canonical 100ps simulation with 2fs timestep was carried out with restraints on heavy atoms, and the temperature was set to 300K using Bussi velocity-rescaling thermostat[14]. Then system was moved to NPT ensemble, and pressure was adjusted 1 bar using Parrinello-Rahman barostat[15]. Timestep was increased to 5fs, and the system was simulated in total 15ns where restraints were reduced gradually from heavy atoms, to backbone and Ca atoms. Particle mesh Ewald (PME) was used with a 1nm cutoff for electrostatics[16]. Neighbor list was updated every 50 step using Verlet cutoff scheme. All bonds were constrained using LINCS algorithm. Production runs were carried out with no restraints under NPT ensemble. Steered MD and accelerated weight histogram (AWH)[17] simulations of RBP were run under the same ensemble as the production runs. Reaction coordinate for these biased simulations were defined as the center-of-masses vectors between two domains. In steered MD, domains were pulled with a rate of 0.0001 nm/ps using a harmonic force constant 8000 kJ mol^{-1} nm^{-2}. In AWH simulation the sampling rate was set to 0.0001 nm^2/ps and sampling interval was limited to 2.8-3.3 nm along the vector.

A simulation of the 4NPQ structure of GLIC in pH 4.6 was setup in united POPC bilayer with virtual sites. Amber99SB-ILDN (protein)[12] and Berger (lipids)[18] force-fields were used. The protonation state of GLIC was chosen based on the calculations of Calimet et al.[19]. Final system contained 306 POPC molecules and ~38600 TIP3P waters. To achieve an ionic strength of 0.1M random water molecules were replaced with 69 Na^+ and 99 Cl^- ions. System

was energy minimized to a tolerance of 1000 kJ/mol/nm with 2fs timestep. Equilibration was carried out under NPT ensemble for 45 ns where restraints were reduced gradually from heavy atoms, to backbone and Ca atoms. Timestep for equilibration was increased to 5fs, temperature was set to 300K using Bussi velocity-rescaling thermostat and pressure was adjusted to 1 bar using semi-isotropic Berendsen weak barostat[20]. For production run all the restraints were removed, pressure coupling was switched to Parrinello-Rahman barostat. Electrostatics were treated with PME using 1nm cutoff and all bonds were constrained using LINCS algorithm.

SUPPLEMENTARY REFERENCES

1. Björkman, J. & Mowbray, S. L. Multiple open forms of ribose-binding protein trace the path of its conformational change. *J. Mol. Biol.* **279,** 651–664 (1998).
2. Schultz-Heienbrok, R., Maier, T. & Sträter, N. Trapping a 96 degrees domain rotation in two distinct conformations by engineered disulfide bridges. *Protein Sci.* **13,** 1811–1822 (2004).
3. Gan, J. *et al.* Intermediate states of ribonuclease III in complex with double-stranded RNA. *Structure* **13,** 1435–1442 (2005).
4. Smolin, N. & Robia, S. L. A structural mechanism for calcium transporter headpiece closure. *J. Phys. Chem. B* **119,** 1407–1415 (2015).
5. Prevost, M. S. *et al.* A locally closed conformation of a bacterial pentameric proton-gated ion channel. *Nature Structural & Molecular Biology* **19,** 642–649 (2012).
6. Sauguet, L. *et al.* Crystal structures of a pentameric ligand-gated ion channel provide a mechanism for activation. *Proc. Natl. Acad. Sci. U. S. A.* **111,** 966–71 (2014).
7. Gu, M. & Rice, C. M. Three conformational snapshots of the hepatitis C virus NS3 helicase reveal a ratchet translocation mechanism. *Proc. Natl. Acad. Sci. U. S. A.* **107,** 521–528 (2010).
8. Appleby, T. C. *et al.* Visualizing ATP-dependent RNA translocation by the NS3 helicase from HCV. *J. Mol. Biol.* **405,** 1139–1153 (2011).
9. Bocquet, N. *et al.* X-ray structure of a pentameric ligand-gated ion channel in an apparently open conformation. *Nature* **457,** 111–114 (2009).
10. Nury, H. *et al.* One-microsecond molecular dynamics simulation of channel gating in a nicotinic receptor homologue. *Proc. Natl. Acad. Sci. U. S. A.* **107,** 6275–80 (2010).
11. Fitzgibbon, A., Pilu, M. & Fisher, R. B. Direct least square fitting of ellipses. *IEEE Trans. Pattern Anal. Mach. Intell.* **21,** 476–480 (1999).
12. Lindorff-Larsen, K. *et al.* Improved side-chain torsion potentials for the Amber ff99SB protein force field. *Proteins* **78,** 1950–1958 (2010).
13. Abraham, M. J. *et al.* GROMACS: High performance molecular simulations through multi-level parallelism from laptops to supercomputers. *SoftwareX* **1–2 IS -,** 19–25
14. Bussi, G., Donadio, D. & Parrinello, M. Canonical sampling through velocity rescaling. *J. Chem. Phys.* **126,** (2007).
15. Nosé, S. & Klein, M. L. Constant pressure molecular dynamics for molecular systems. *Mol. Phys.* **50,** 1055–1076 (1983).
16. Essmann, U. *et al.* A smooth particle mesh Ewald method. *J Chem Phys* **103,** 8577–8593 (1995).
17. Lindahl, V., Lidmar, J. & Hess, B. Accelerated weight histogram method for exploring free energy landscapes. *J. Chem. Phys.* **141,** 044110 (2014).
18. Berger, O., Edholm, O. & Jähnig, F. Molecular dynamics simulations of a fluid bilayer of dipalmitoylphosphatidylcholine at full hydration, constant pressure, and constant temperature. *Biophys. J.* **72,** 2002–2013 (1997).
19. Calimet, N. *et al.* A gating mechanism of pentameric ligand-gated ion channels. *Proc. Natl. Acad. Sci. U. S. A.* **110,** E3987–96 (2013).
20. Berendsen, H. J. C., Postma, J. P. M., van Gunsteren, W. F., DiNola, a & Haak, J. R. Molecular dynamics with coupling to an external bath. *J. Chem. Phys.* **81,** 3684–3690 (1984).
