# Supplementary material for: Prediction and validation of protein intermediate states from structurally rich ensembles and coarse-grained simulations
Source: Nat Commun. 2016 Aug 31;7:12575. doi: 10.1038/ncomms12575 (PMC5013691; doi:10.1038/ncomms12575)
Supplement: Supplementary Data 1 — Supplementary .html file containing interactive PC1-2 plots with links to the Protein Data Bank [file ncomms12575-s2.zip › interactiveplots/Transitions.html]

Transitions

 

### Graph settings

Select protein:
RBP
5Nase
RNase III
SERCA
GLIC

Reset zoom

### eBDIMS transition pathways

Forward  
Reverse

### Data
